# Nanopods: A New Bacterial Structure and Mechanism for Deployment of Outer Membrane Vesicles

**DOI:** 10.1371/journal.pone.0020725

**Published:** 2011-06-07

**Authors:** Ameesha Shetty, Shicheng Chen, Elitza I. Tocheva, Grant J. Jensen, William J. Hickey

**Affiliations:** 1 Molecular and Environmental Toxicology Program, University of Wisconsin-Madison, Madison, Wisconsin, United States of America; 2 O.N. Allen Laboratory for Soil Microbiology, Department of Soil Science, University of Wisconsin-Madison, Madison, Wisconsin, United States of America; 3 Division of Biology, California Institute of Technology, Pasadena, California, United States of America; 4 Howard Hughes Medical Institute, California Institute of Technology, Pasadena, California, United States of America; University of Osnabrueck, Germany

## Abstract

**Background:**

Bacterial outer membrane vesicles (OMV) are packets of periplasmic material that, *via* the proteins and other molecules they contain, project metabolic function into the environment. While OMV production is widespread in proteobacteria, they have been extensively studied only in pathogens, which inhabit fully hydrated environments. However, many (arguably most) bacterial habitats, such as soil, are only partially hydrated. In the latter, water is characteristically distributed as films on soil particles that are, on average thinner, than are typical OMV (*ca*. ≤10 nm water film *vs.* 20 to >200 nm OMV;).

**Methodology/Principal Findings:**

We have identified a new bacterial surface structure, termed a “nanopod”, that is a conduit for projecting OMV significant distances (*e.g*., ≥6 µm) from the cell. Electron cryotomography was used to determine nanopod three-dimensional structure, which revealed chains of vesicles within an undulating, tubular element. By using immunoelectron microscopy, proteomics, heterologous expression and mutagenesis, the tubes were determined to be an assembly of a surface layer protein (NpdA), and the interior structures identified as OMV. Specific metabolic function(s) for nanopods produced by *Delftia* sp. Cs1-4 are not yet known. However, a connection with phenanthrene degradation is a possibility since nanopod formation was induced by growth on phenanthrene. Orthologs of NpdA were identified in three other genera of the *Comamonadaceae* family, and all were experimentally verified to form nanopods.

**Conclusions/Significance:**

Nanopods are new bacterial organelles, and establish a new paradigm in the mechanisms by which bacteria effect long-distance interactions with their environment. Specifically, they create a pathway through which cells can effectively deploy OMV, and the biological activity these transmit, in a diffusion-independent manner. Nanopods would thus allow environmental bacteria to expand their metabolic sphere of influence in a manner previously unknown for these organisms.

## Introduction

The ability of bacteria to extend their sphere of metabolic influence long distances (*ca*. microns) from the cell is key to their activity and survival, and is achieved by secretion of small molecules, such as acyl homoserine lactones [1], [2], which can have broad, regulatory effects on the metabolism of neighboring bacteria, as well as macromolecules, namely enzymes and outer membrane vesicles (OMV), which transmit specific function(s) [3]. The latter are unique in that they can encompass a broad range of (macro)molecules, which mediate a variety of processes. For example, OMV can package small molecules for signaling [4] and proteins that effect virulence [3], [5], [6] *;* OMV-mediated DNA transfer has also been demonstrated [7]. These vesicles are highly versatile as they can be designed for different functions by different organisms, and tasked for different activities by the same organism [8]. Thus, OMV are a type of bacterial “Swiss army knife” for projecting extracellular activities and, perhaps reflecting their utility, their production is widespread in proteobacteria [5], [9], [10]. But, despite their prominence, the biology of OMV has been extensively studied only in pathogens, for which these are key vehicles for long distance transmission of virulence factors [11], [12], [13].

A fundamental feature of OMV deployment is the dependence on diffusion and, consequently, the environment's hydration status. In this regard, a fully hydrated environment (water replete), such as that experienced by pathogens in their host, allows diffusive movement that is effectively non-restricted. However, many (arguably most) bacterial habitats, such as soil, are only partially hydrated. In soil, water is characteristically distributed as films on particles that are, on average, estimated to be thinner than are typical OMV (*ca*. ≤10 nm water film [14] *vs.* 20 to >200 nm OMV [3], [5]). Partial hydration is also restrictive in that a capillary pinning force may arise that, as the name suggests, would cause OMV to adhere to surfaces of soil particles [15]. Conditions in soil that would be conducive to effective movement by diffusion would likely be limited to relatively brief periods following large influxes of water, such as a heavy rain. The question then, is: Are environmental bacteria (*e.g.*, soil bacteria) unable to utilize OMV to externally expand their sphere of metabolic influence in a manner akin to pathogens, or do they possess diffusion-independent mechanisms for OMV deployment and, if latter, what are they?

The present report describes novel bacterial organelles, termed “nanopods”, that can project OMV long distances (≥6 µm) from the cell. Nanopod deployment of OMV is independent of diffusion, and thus represents a solution to constraints imposed by partial hydration and, consequently, a new paradigm in the mechanisms of long distance interaction utilized by bacteria.

## Results and Discussion

**Figure 1:**
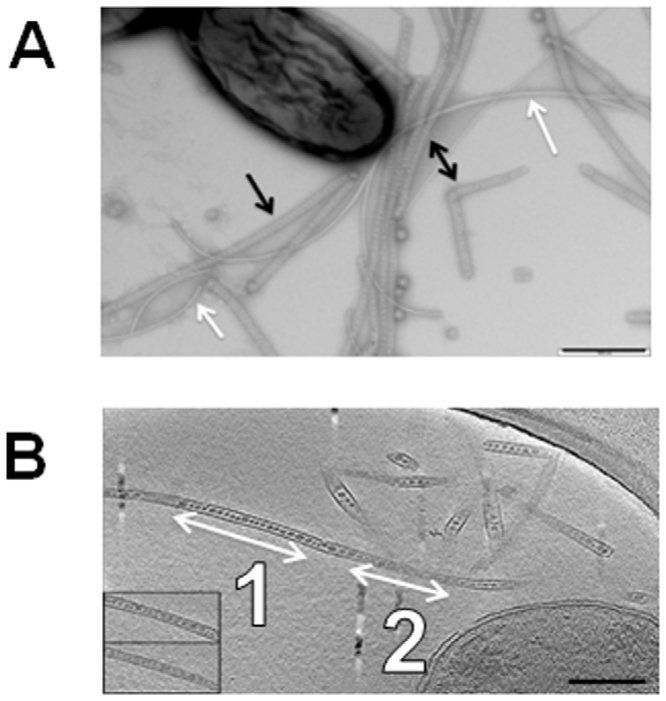
Images of nanopods in phenanthrene-grown culture of strain Cs1-4. Panel A. Negatively stained wet mount of a phenanthrene-grown culture of strain Cs1-4 showing nanopods (black arrows) and flagella (white arrows); scale bar  = 500 nm. Panel B. Central slice through a 3-D electron cryotomographic reconstruction of a phenanthrene-grown *Delftia* sp. Cs1-4 culture showing nanopods next to a cell. Arrows follow an individual nanopod, and illustrates its undulating structure. Regions denotated as “1” are the exposed interior with vesicles. Areas indicated by “2” are the outer surface. Insets: Tomographic slices just above and below the region labeled “1”, showing the surface and the crystalline array. Scale bar  = 200 nm.

Nanopods were discovered in phenanthrene-grown cultures of *Delftia* sp. Cs1-4, a polycyclic aromatic hydrocarbon (PAH)-degrading bacterium that was isolated from PAH-contaminated soil in Wisconsin [16]. Imaging of phenanthrene-grown batch (shaken) cultures of strain Cs1-4 by transmission electron microscopy (TEM) revealed an abundance of detached structures (up to 6 µm in length) that had a crystalline-like outer surface, and contained interior structures that varied in morphology from spherical to spiral (Fig. 1A, fig. S1A). Notably, the outer surface structure of these particles resembled the crystalline surface layer that covers cells of *Delftia* sp. Cs1-4, as well as its close relative, *Delftia acidovorans* ATCC15688 [17]. TEM-Imaging of nanopods in thin sections also showed interior vesicle-like structures, which were contained within an encasing structure (fig. S1B,C). Electron cryotomography images were consistent with those from TEM in exposing the crystalline-like outer layer and the internal vesicle-like elements (Fig. 1B). Furthermore, three-dimensional images constructed from electron cryotomography, revealed nanopods to be have an undulating tubular architecture unlike the linear, filamentous construction characteristic of flagella or pili (Fig. 1B; Movie S1).

**Figure 2:**
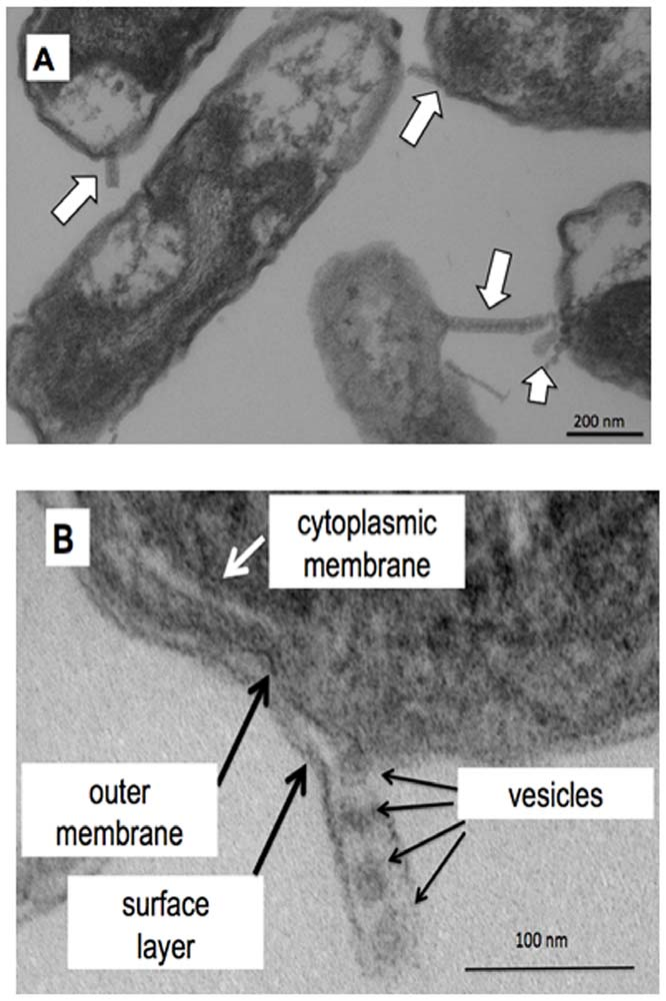
Cell-attached nanopods in phenanthrene-grown biofilm cells of *Delftia* sp. Cs1-4. Panel A. Thin section of phenanthrene-grown biofilm cells of *Delftia* sp. Cs1-4 showing cell-attached nanopods (white arrows). Scale bar  = 200 nm. Panel B. Detail view of a cell-nanopod junction. Scale bar  = 100 nm.

The native structure of nanopods was observed in biofilm cultures, which were grown statically on phenanthrene-coated glass cover slips. Nanopods projecting from cell surfaces were abundant and often spanned the space between neighboring bacteria (Fig. 2A). Nanopods appeared to arise from various (and sometimes multiple) positions on the cells, but there was some tendency toward the polar sections. With their native structure intact, the nanopod outer layer was contiguous with a surface layer on the cell (Fig. 2B), and association of the nanopod's interior vesicle-like structures with the outer membrane was discernable (Fig. 2B). The biofilm images showed that nanopods were out-growths from the cell surface, and allowed us to formulate an hypothesis regarding the structure and composition of nanopods. We hypothesized that the internal structures in nanopods were outer membrane vesicles (OMV) and the encasing structure was the cell surface layer protein (SLP).

Testing of the hypothesized structure of nanopods was conducted along two paths. In one of these, the possibility that the interior structures were OMV was evaluated by examining nanopods for biomolecules that are unique to the OM, namely lipopolysaccaride (LPS) and outer membrane proteins (OMP). The other track, focused on identifying the SLP and ascertaining its potential role as the encasing structure *via* mutagenesis and immunoelectron microscopy.

**Figure 3:**
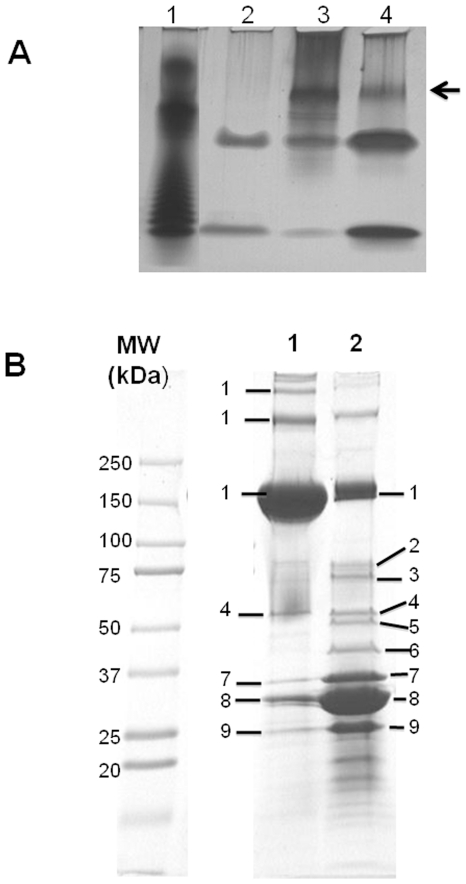
Analysis of lipopolysaccharide and proteins in nanopods and outer membrane. Panel A. Analysis of lipopolysaccaride (LPS) by tricine-SDS-PAGE and silver staining. Samples of LPS were extracted from: purified nanopods (Lane 2), *Delftia* sp. Cs1-4 cells grown on phenanthrene (Lane 3) and *Delftia* sp. Cs1-4 cells grown on pyruvate (Lane 4). Lane 1 is an LPS standard *(Salmonella typhimurium).* The arrow indicates a dense band of LPS present in whole cell preparations but absent from nanopods. All samples were loaded at the same dry weight (200 µg). Panel B. SDS-PAGE Protein profiles of nanopods (Lane 1) and the outer membrane (OM) of phenanthrene-grown *Delftia* sp. Cs1-4 cells (Lane 2). Proteins identified in gel slices from the OM sample are (locus in *Delftia* sp. Cs1-4): 1. NpdA (DelCs14_2799), 2. Fiu-like TonB-dependent siderophore receptor (DelCs14_0908), 3. TonB-dependent siderophore receptor (DelCs14_5618) 4. Protein with domain of unknown function 1302 (DelCs14_4425), 5. RND efflux system, outer membrane lipoprotein (DelCs14_5845) 6. 4. Type II L-Asparaginase, 7. OmpC-like protein (DelCs14_0125), 8. Omp32 (DelCs14_0124). 9. OmpA-like protein (DelCs14_3211).

The LPS analysis verified the presence of these molecules in nanopods, and also indicated that the composition of LPS in nanopods differed from that of whole cells (Fig. 3A). If, as we hypothesize, the internal structures are OMV, the divergence in LPS content of nanopods *vs.* cells would be similar to that noted in comparisons of OMV *vs.* cells of other bacteria, including *Pseudomonas aeruginosa* [18], *Porphyromonas gingivalis* [19] and *Xanthomonas campestris* [20]. Variation in LPS content of OMV has been proposed to have a role in biogenesis of these structures [18], [20] and, if true, could have a role in nanopod formation as well.

Nanopod protein content was analyzed by two different approaches: Mass spectrometry (MALDI-TOF/TOF) of individual proteins separated by SDS-PAGE profiles (Fig. 3B) and “bottom-up” mass spectrometry (LC-MS/MS) of whole nanopod preparations (Table 1). In the former, nanopods were highly enriched in a series of high molecular weight bands (≥150 kDa) that were all identified as the same “hypothetical” protein in the genome of *Delftia* sp. Cs1-4. Because of its abundance in nanopods, this protein was termed Nanopod protein A (NpdA). Other proteins prominent in nanopod's profile were OMP, or proteins predicted to be secreted (Fig. 3B). Bottom-up analysis of nanopods yielded consistent identification (*e.g*., in three biological replicates) of eighteen proteins that could be separated into three groups (Table 1): Structural proteins associated with the cell surface or OM, periplasmic enzymes and proteins of unknown function. Notably absent from any protein analysis of nanopods were proteins known to be either cytoplasmic, or associated with the cytoplasmic membrane.

**Table 1:** Proteins identified in whole nanopod samples.^1^

Category	
Cell surface/outer membrane	Locus^2^
Surface layer protein (NpdA)^3^	DelCs14_5206
Ig Family protein	DelCs14_1039
Omp32	DelCs14_0919
OmpA/MotB Domain	DelCs14_2139
Outer membrane protein assembly complex	DelCs14_1911
TonB-dependent siderophore receptor	DelCs14_2104
TonB-dependent siderophore receptor	DelCs14_5255
TonB-dependent siderophore receptor	DelCs14_5519
Transport-associated protein	DelCs14_5457
VacJ Family lipoprotein	DelCs14_1081
Periplasmic enzymes	
γ-Glutamyltransferase	DelCs14_5097
Peptidase M30	DelCs14_0216
Peptidase S45	DelCs14_3342
Hydroxybutyrate-dimer hydrolase	DelCs14_4099
Unknown	
Hemolysin coregulated protein (Hcp)	DelCs14_2985
TPR repeat-containing protein	DelCs14_3801
Conserved hypothetical protein	DelCs14_4061
Unknown function (DUF1302)	DelCs14_1756
Unknown function (DUF1329)	DelCs14_1757

1 Identified in three independent samples.

2 Numbering in *Delftia* sp. Cs1-4 genome.

3 Nanopod protein A, see text.

The forgoing provided strong evidence supporting our hypothesis, inferred from electron microscopy images, that the structures contained within nanopods are OMV. It can be argued, however, that identification of LPS or OMP does not directly provide information as to the origin of these molecules or their physical location in nanopods, and as such the origin and physical association of these molecules cannot be inferred from the biochemical analyses. While such alternate interpretations cannot be unequivocally ruled out, they are substantially weakened on two accounts. First, a great body of literature exists that shows OMP and LPS to be unique to the OM, and thus reliable biomarkers for tracking OM-derived materials. Second, the physical behavior of these molecules is well established, specifically, the formation of bilayer membranes/micelles by LPS, and the integration of OMP into such membranes/micelles. Furthermore, the alternative interpretation leads to no alterative hypothesis as to the nature of the internal structures. Thus, on balance, we favor interpretation of the above-summarized data as indicative of OMV.

The other track of the project focused on analysis of NpdA as a candidate for the SLP. The gene encoding NpdA was cloned from *Delftia* sp. Cs1-4, and its expression in *E. coli* gave a product of the predicted size (ca. 61 kDa), which was used for antibody production. In Western blots of whole cell protein, antibody bound only to the >150 kDa bands; no band corresponding to the monomer was detected (fig. S2A). These findings suggested that NpdA exists natively as an oligomer, and that the oligomeric form was not effectively disrupted by SDS.

**Figure 4:**
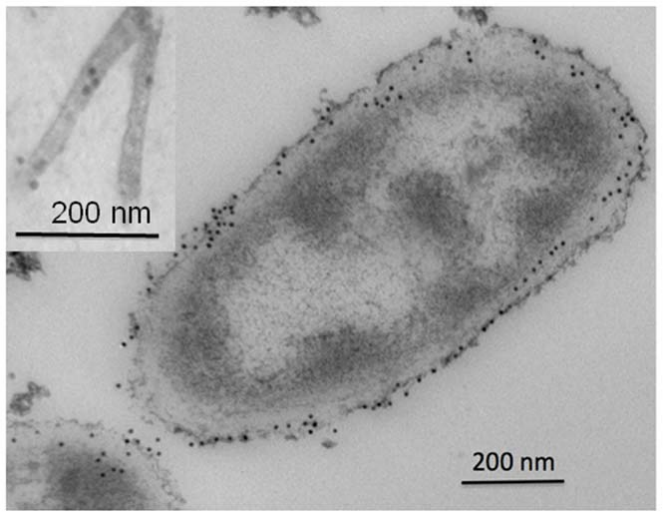
Immunogold labeling of whole cells of *Delftia* sp. Cs1-4 and nanopods (inset) showing location of NpdA as indicated by 10 nm gold particles (black dots).

Localization of NpdA was done *via* immunogold labeling experiments with anti-NpdA. For whole cells, label was restricted to the envelope region, as would be expected for an SLP (Fig. 4). The pattern and extent of labeling with anti-NpdA was similar to that attained in other immuno-microscopy studies of SLP [21]. Nanopods were also labeled by anti-NpdA, confirming the presence of NpdA in these structures. It's important to note that, although NpdA appeared from SDS-PAGE analyis to be a major component of nanopods, labeling of these structures more extensive than that observed was not necessarily expected. This is because the extent of labeling achieved in immunoelectron microscopy is well-known to be affected by many variables associated with sample processing [22], and with variation in epitopes exposed during antibody generation *vs.* those exposed by the protein in its native state (*e.g*., as an oligomer in the case of NpdA). For nanopods, epitope exposure would have also been affected by their undulating structure (Fig. 1B, Movie S1), which would have limited substrate exposure (*e.g.*, that exposed by sectioning in a single plane) to relatively short segments of these structures. The latter variable would likely reduce the extent labeling (*e.g.*, relative to whole cells) as suitable epitopes may or may not be exposed in these areas.

**Figure 5:**
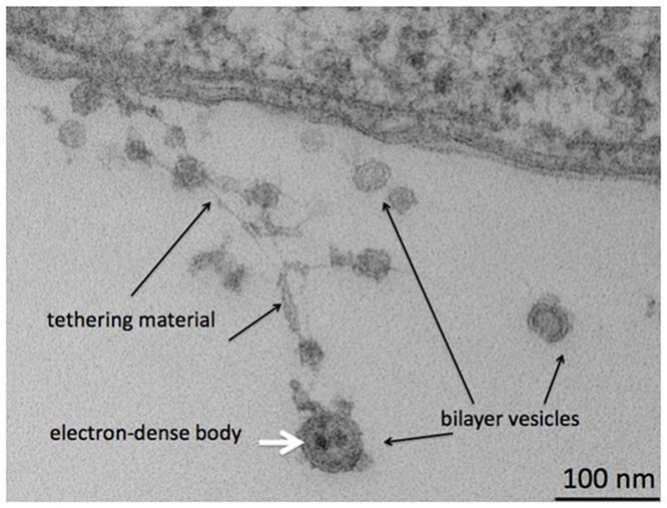
Thin section of the NpdA mutant of *Delftia* sp. Cs1-4 showing absence of S-layer (cf. Fig. 2B) and extra-cellular vesicles.

The *npdA* gene was disrupted by allelic exchange, and protein profiles of the mutant (strain M3) lacked the bands from which NpdA was identified (fig. S2A). The protein was also not detected in Western blots of the mutant (fig. S2A). In thin sections imaged by TEM, the S-layer was no longer visible in the mutant (Fig. 5), and in immuno-electron microscopy the mutant cells showed no labeling with anti-NpdA. Inspection by TEM of the NpdA mutant confirmed the absence of nanopods (figs. S2B,C). The mutant did however, continued to produce OMV, which were observed in TEM images as tethered, extracellular bilayer vesicles (Fig. 5). Lastly, the effect of the loss of NpdA on nanopod formation was assessed quantitatively by using anti-NpdA in fluorescent antibody analysis, which showed non-detectable levels of nanopods in phenanthrene-grown cultures of the mutant.

The foregoing established NpdA as a surface associated protein, which is essential for both the formation of the S-layer and nanopods. Our hypothesis is that NpdA is the protein constituting the surface layer in *Delftia* sp. Cs1-4; this hypothesis is based on data from the present study and that of Engelhardt *et al*. [17]. The latter investigators demonstrated that a single polypeptide formed the tetragonal lattice S-layer of *Delftia* (formerly *Comamonas*) *acidovorans* ATCC15688. Engelhardt and coworkers did not report the amino acid sequence for the *D. acidovorans* SLP. But, in our analysis of *D. acidovorans* ATCC15688 (see below), the protein studied by the Engelhardt group was determined by mass spectrometry to be an NpdA ortholog.

Collectively, the data summarized above supported our hypothesized structure of nanopods. Specifically, these were chains of OMV encased within an SLP. Engelhardt *et al*. [17] did not report nanopod-like structures. However, in their reconstitution experiments with SLP (NpdA) extracted from cells, cylindrical or sheet-like structures could be formed, but their assembly required the addition of LPS. A defining characteristic of most SLP is their ability to self-assemble (*i.e*., independently of other molecules) into sheets and other structures [23]. Thus, the *D. acidovorans* SLP (NpdA) differed from other SLP in that its ability to self-assemble was LPS-dependent. We hypothesize that the strong association of LPS with NpdA could play a key role in the assembly and stabilization of the nanopod structure. In this respect, we can envision that as OMV pinch off from the cell surface, a strong LPS-NpdA association could be an important factor enabling retention of the S-layer coating by OMV. Thus, an OMV chain contained within the S-layer could form as a series of OMV pinch off from a specific point.

Specific metabolic function(s) for nanopods produced by *Delftia* sp. Cs1-4 are not yet known. However, a connection with phenanthrene degradation is a possibility since nanopod formation was induced by growth on phenanthrene. This effect was determined by using anti-NpdA in fluorescent antibody analysis, which showed nanopod levels in phenanthrene-grown cultures were *ca.* six-fold greater than in cultures grown on pyruvate, succinate or vanillate. It's also notable that genes for two of the proteins identified in nanopods (DUF 1329 and DUF 1302) are located adjacently within the gene cluster encoding phenanthrene degradation.

While metabolic activities of nanopods remain to be determined, their structure can be perceived to confer unique benefits on a producer inhabiting a partially hydrated environment, such as soil. Specifically, in the case of *Delftia* sp. Cs1-4, the OMV it produced contained an array of enzymes, and nanopod deployment of OMV would allow the cell to circumvent constraints imposed by diffusion, and utilize OMV for long-distance projection of metabolic activity. Nanopods would thus represent a unique ecological adaptation, which, to the best of the authors' knowledge, has not been previously reported.

The deduced amino acid sequence of NpdA was 50 to 62% similar (positive residues, 96 to 99% coverage) to orthologs in the genomes of five bacteria from genera of the *Comamonadaceae*: *Acidovorax avenae s*ubsp. *avenae* ATCC 19860, *A. avenae s*ubsp. *citrulli* AAC00-1, *A. delafieldii, Delftia acidovoran*s SPH-1 and *Verminephrobacter eiseniae* EF01-2. The cognate gene was present in a single copy in the genomes of all of these organisms except strains AAC00-1 (two copies) and ATCC 19860 (four copies). Cultures of all of the above-described bacteria, except strain *A. avenae s*ubsp. *avenae* ATCC 19860 (unavailable), were examined by TEM and confirmed to contain nanopods (figs. S3A–C,E). The type strain *D. acidovoran*s ATCC 15688 also produced nanopods (fig. S3D); *D. acidovoran*s ATCC 15688 currently lacks genome sequence data, but its possession of a NpdA ortholog was verified in this study by mass spectrometry of an SDS-PAGE gel-band. Thus, nanopod formation appears to be a characteristic of NpdA-producing bacteria, which so far are a subgroup of the *Comamonadaceae.*

Bacteria that to date are known to produce nanopods have diverse lifestyles. *Delftia* sp. Cs1-4, *D. acidovorans* SPH1 and *A. delafieldii* are similar in that they are both free-living soil bacteria. In contrast, *A. avenae s*ubsp. *avenae* ATCC 19860, *A. avenae* subsp. *citrulli* and *V. eiseniae* are biotrophs, and live associated with eukaryotic organisms. The former two are plant pathogens, while *V. eiseniae* EF01-2 is an earthworm symbiont, and was isolated from nephridia of *Eisenia foetida* [24], which inhabits environments of decomposing organic material. It will be interesting to determine how, or if, bacteria tailor nanopods for unique functions in each of these habitats.

## Materials and Methods

### Culture growth

*Delftia* sp. Cs1-4 was grown in defined mineral salts medium (MSM, [25]) with either phenanthrene (Sigma-Aldrich, 98%) or pyruvate (Sigma-Aldrich, 99%) as the sole carbon and energy source. Phenanthrene and pyruvate were added to 50 mM on an electron equivalent basis. For growth of biofilms, phenanthrene-coated, glass cover-slips were floated overnight in petri dishes containing a phenanthrene-grown liquid culture of *Delftia* sp. Cs1-4 to allow cell attachment. Cover slips were then transferred to petri dishes containing fresh MSM, covered with foil and incubated statically for 14 d. Cover slips were then removed, and processed for TEM as described below. For electron microscopy, cultures of *Acidovorax avenae s*ubsp. *avenae* ATCC 19860, *A. avenae s*ubsp. *citrulli* AAC00-1, *A. delafieldii, Delftia acidovoran*s SPH-1 and *D. acidovorans* ATCC 15688 were grown on Nutrient Broth (Difco). *Verminephrobacter eiseniae* EF01-2 was cultured in *Acidovorax* complex medium [24]. Genbank accession numbers for NpdA orthologs: *Delftia* sp. Cs1-4; *D. acidovorans* SPH1, YP_001562340.1; *A. delafieldii*, ZP_04761493.1; *A. avenae s*ubsp. *citrulli* AAC00-1, YP_972463.1, YP_972802.1; *A. avenae s*ubsp. *avenae* ATCC 19860, ZP_06211954.1, ZP_06211817.1 ZP_06210145.1; *V. eiseniae* EF01-2, YP_995476.1.

### Preparation of nanopods

Nanopods were harvested from culture fluids of a strain Cs1-4 mutant that is devoid of flagella production. Cell removal was done by centrifugation (20 min, 6,000×*g*) followed by filtration (2X passage through 0.2 µM pore diam membrane). Nanopods in the filtrate were pelleted (60 min. 15,000×*g*), resuspended in ddH_2_0, dialyzed against ddH_2_0 and then freeze-dried. Purified preparations were confirmed to be cell-free by visual inspection (TEM) as well as by inoculating samples into liquid MSM-phenanthrene. For the latter, no growth was observed after >12 wk of incubation.

### Transmission electron microscopy

Ultrastructure of biofilms was examined by using a “pop-off” technique. Briefly, biofilm-containing coverslips were fixed with 2.5% glutaraldehyde, post-fixed in OsO_4_ and then processed through a graded series of alcohols. Capsular molds containing liquid epoxy resin are then placed inverted on the biofilms, and following polymerization, the molds were “popped-off”. The blocks were then thin-sectioned, and stained with uranyl acetate and lead citrate. Negative staining was done by mixing samples of culture fluids with 2.0% phosphotungstic acid. All samples were imaged with a Philips CM120 scanning transmission electron microscope.

### Antibody production, Western blotting, Immunoelectron microscopy and fluorescent antibody assay

Proteins targeted for antibody production was expressed in *E. coli* strain BL21-AI by using Pet5a system (0.2% arabinose induction). Whole cell protein was separated by 4–20% SDS-PAGE, and the protein (1 mg) purified, concentrated and emulsified with Freund's adjuvant and injected into hens. A booster injection of the same amount was administered 15 days later. Egg collection began 7 d after the booster, and antibody titers peaked within 3 weeks. For antibody preparation, egg yolks were harvested, and IgY precipitated with PEG 6000 [26].

For Western blotting, protein extracts (50 µg each) were separated by SDS-PAGE and then transferred (30 V, 1 h) to PVDF membranes by using a Mini Trans-blot (Biorad). The membranes were blocked by overnight incubation in a solution of 5% evaporated milk in 1X Tris buffered saline Tween 20 (TBST). Membranes were then probed with IgY (1∶10,000) for one hour, followed by three washes with TBST for 10 min each. Secondary labeling was done with goat anti-chicken antibody (Bethyl laboratories) at a dilution of 1∶20,000 for one hour. Detection was done by using super signal west pico chemiluminescent substrate (Thermo-Scientific) according to manufacturer's protocol; films were developed for 2 min each.

For immunoelectron microscopy, specimens were fixed by freeze-substitution, and embedded in Lowicryl HM20**.** Sections were labeled with anti-NpdA, and then with anti-IgY conjugated to gold particles (10 nm diam). For negative controls, thin sections were exposed only to secondary antibody.

Nanopods were quantified by using a fluorescent antibody assay. Nanopods were prepared as described above. The pellet obtained was resuspended in 500 µL phosphate buffer (pH 7.0) and labeled with anti-NpdA antibody (1∶1000) at 37 °C for 10 minutes. This was followed by centrifugation at 20,000×*g* for 1 h. After two washings with sterile phosphate buffer, it was labeled with the secondary antibody (1∶500) Dy-Light 488 anti IgY conjugate (Jackson Immunoresearch) for 10 min at 37 °C. This step was followed by centrifugation at 20,000×*g* for 1 h followed by two more washes with sterile phosphate buffer. The final pellet was resuspended with 200 µL phosphate buffer, and fluorescence was quantified using a Synergy microplate reader (Biotek, VT). Cultures grown on phenanthrene and pyruvate were tested during early (1 d) and late (7 d) stages of their growth phase. Samples taken at these time points were also centrifuged at 20,000×*g* for 1 h, and visualized by using negative staining (Uranyl acetate) transmission electron microscopy (TEM).

### Electron cryotomography

Samples were mixed with 10 nm colloidal gold particles (BB International), loaded onto glow-discharged lacy carbon grids (Ted Pella, Inc.) and quick-frozen by plunging into liquid ethane with a FEI Mark III Vitrobot and imaged with a Tecnai Polara, which is a 300 kV TEM. Tilt series (±60°, 1.5° angular increments) were digitally recorded on the GIF CCD using the UCSF tomography software package [27] on a 300 kV, FEG, G2 Polara transmission EM (FEI). Images were acquired under low-dose conditions (80–100 e^−^/Å^2^ total for the tilt series) 6–8 µm underfocus (first CTF zero at 3.5–4.0 nm) at 34,000 X, such that each pixel represented 6.8 Å. In some cases, dual-axis tilt series were recorded by rotating the grid 90° [28].

### Lipopolysaccharide (LPS) analysis

LPS was extracted from phenanthrene- and pyruvate-grown *Delfita* sp. Cs1-4 cells, as well as from nanopods, by using the Tri method described by Yi and Hackett (Yi, 2000). Freeze-dried material (5–10 mg) was dissolved in Tri-reagent, followed by five extractions with water, and the pooled aqueous fractions were dried with a speed vac. The dried material was then resuspended in 100 µL of 375 mM MgCl_2_ in ethanol. The white pellet obtained after centrifugation at 10,000×*g* was dissolved in 200 µL water, and freeze-dried to obtain a white fluffy solid (LPS), which was used for further analysis. The LPS samples (400 µg) were run using tricine-SDS PAGE on a tricine-tris gel (10–20% continuous gradient, Biorad) at 30 mA for 5 h, followed by 90 mA for 2 h. The gel was stained by using a Silver staining kit (Biorad). LPS of *S. typhimurium* (Sigma) was loaded (50 µg) of as a standard.

### Outer membrane preparation

Outer membranes were prepared by following the method of Frias *et al*. [9]. Cells of *Delftia* sp Cs1-4 were harvested at late log phase from a phenanthrene-grown culture (500 mL) by centrifugation (10,000×*g*, 10 min). The pellet was washed twice with 10 mM HEPES buffer (pH 7.4) and then resuspended in 5 mL of the same buffer. The cell suspension was lysed *via* sonication by using a Braun Sonic 2000 U. The sample was placed on ice, and then subjected to 6 pulses (30 W, 1 min each) *via* a needle probe (40 T, Braun). The lysate was then centrifuged (10,000×*g*, 10 min) to remove cellular debris, and the supernatant clarified by passage through a 0.2 µm syringe filter. The filtrate was subjected to centrifugation at 40,000×*g* for 60 min (4°C) in an JA-20 rotor (Beckman Coulter) housed in a Avanti J-E centrifuge (Beckman Coulter). The crude membrane pellet was resupended in 2% (w/v) Sarkosyl (Sigma) in 10 mM HEPES buffer (pH 7.4) and incubated at room temperature for 30 min to solubilize the inner membrane. The suspension was then centrifuged at 40,000×*g* for 90 min (20°C), and the resulting pellet was resuspended in 10 mM HEPES buffer for use in further analyses.

### Proteomics

For identification of proteins comprising bands on SDS-PAGE gels, bands of interest were excised, destained and subjected to a surfactant (ProteasMax, Promega) enhanced in gel digestion procedure. Peptides extracted from the gel were purified by passage over a ZipTip-C18 column (Millipore) and then analyzed by MALDI-TOF/TOF (Applied Biosystems/MDS SCIEX 4800 MALDI TOF/TOF). Tryptic digests of whole samples of nanopods and OM were analyzed by LC-MS/MS (Thermo Scientific LTQ Orbitrap XL). All mass spectral data from was searched against the genome of *Delftia* sp. Cs1-4 (5932 entries) by using Mascot (Matrix Science, London, UK; version Mascot). For MALDI, Mascot was searched assuming the digestion enzyme trypsin, a fragment ion mass tolerance of 0.60 Da and a parent ion tolerance of 15 ppm. Oxidation of methionine was specified in Mascot as a variable modification. Scaffold (version Scaffold_3_00_03, Proteome Software Inc., Portland, OR) was used to validate MS/MS based peptide and protein identifications. Peptide identifications were accepted if they could be established at greater than 95.0% probability as specified by the Peptide Prophet algorithm [29]. Protein probabilities were assigned by the Protein Prophet algorithm [30]. Proteins that contained similar peptides and could not be differentiated based on MS/MS analysis alone were grouped to satisfy the principles of parsimony.

### Mutagenesis

Targeted mutagenesis of *npdA* was done *via* allelic exchange. A *ca*. 1.2 kb of *npdA* was amplified and inserted into the suicide vector pJK100. The construct was conjugatively introduced into strain Cs1-4, and insertion in the targeted location was confirmed by PCR and sequencing of the targeted region.

## Supporting Information

Figure S1 **Panel A. Negative stain of individual nanopods.** Note the crystalline-like outer surface. Also note a point (indicated by arrow) where the interior structure transitions from spherical to a spiral form. Panel B. Longitudinal section of a nanopod showing a single outer layer surrounding internal vesicle-like structures (example indicated by arrow). Panel C. Thin-sectioned samples of nanopods (arrows) cut in cross-section (box) or oblique angles.
(TIF)

Figure S2 **Panel A. Analysis of NpdA in** ***Delftia*** **sp. Cs1-4 wild type (Lanes 1 and 3) and NpdA mutant (Lanes 2 and 4) cells.** Lanes 1–2 are SDS-PAGE profiles; Lanes 3 and 4 are Western blots of whole cell SDS-PAGE profiles probed with anti-NpdA. Panel B. Negatively stained culture of wild type *Delftia* sp. Cs1-4 showing nanopods. Panel C. Negatively stained culture of NpdA mutant *Delftia* sp. Cs1-4 illustrating absence of nanopods.
(TIF)

Figure S3 **Negatively stained culture fluid samples containing nanopods from other** ***Comamonadaceae*** **bacteria.** Nanopods are indicated by thick arrows, thin arrows point to other apparent S-layer-derived structures (indicated by crystalline surface). Samples are from:, *A. avenae s*ubsp. *citrulli* AAC00-1 (Panel A), *A. delafieldii* (Panel B), *D. acidovoran*s SPH-1 (Panel C), *D. acidovorax* ATCC 15688 (Panel D) and *V. eiseniae* EF01-2 (Panel E).
(TIF)

Movie S1 **Three-dimensional reconstruction of a phenanthrene-grown culture of Delftia sp. Cs1-4 imaged by electron cryotomography.** The sample is supported on a lacy carbon grid (outer circle) and contains a cell (inner circular structure) and nanopods (undulating structures in the extracellular space).
(MOV)
