# Supplementary figures and images for: Nanopods: A New Bacterial Structure and Mechanism for Deployment of Outer Membrane Vesicles
Source: PLoS One. 2011 Jun 7;6(6):e20725. doi: 10.1371/journal.pone.0020725 (PMC3110197; doi:10.1371/journal.pone.0020725)

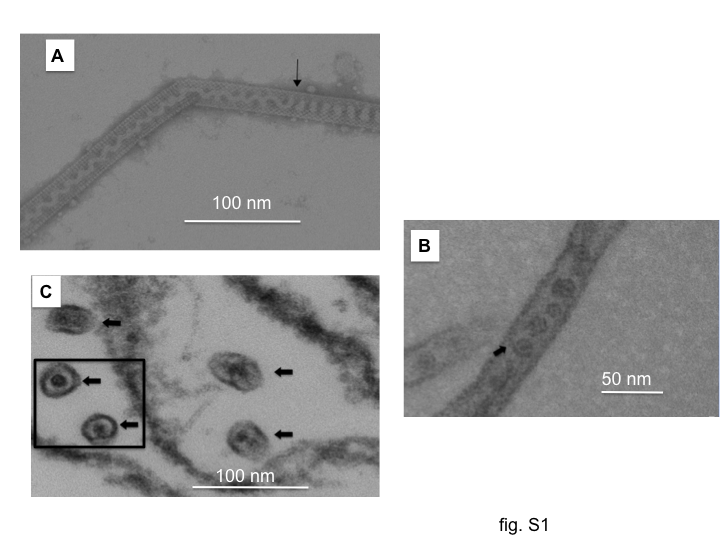

Supplement: Figure S1 — Panel A. Negative stain of individual nanopods. Note the crystalline-like outer surface. Also note a point (indicated by arrow) where the interior structure transitions from spherical to a spiral form. Panel B. Longitudinal section of a nanopod showing a single outer layer surrounding internal vesicle-like structures (example indicated by arrow). Panel C. Thin-sectioned samples of nanopods (arrows) cut in cross-section (box) or oblique angles. (TIF) [file pone.0020725.s001.tif]

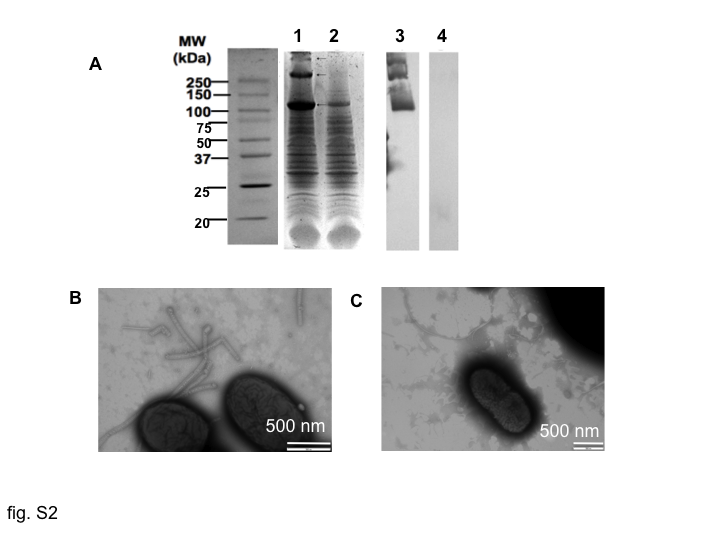

Supplement: Figure S2 — Panel A. Analysis of NpdA in Delftia sp. Cs1-4 wild type (Lanes 1 and 3) and NpdA mutant (Lanes 2 and 4) cells. Lanes 1–2 are SDS-PAGE profiles; Lanes 3 and 4 are Western blots of whole cell SDS-PAGE profiles probed with anti-NpdA. Panel B. Negatively stained culture of wild type Delftia sp. Cs1-4 showing nanopods. Panel C. Negatively stained culture of NpdA mutant Delftia sp. Cs1-4 illustrating absence of nanopods. (TIF) [file pone.0020725.s002.tif]

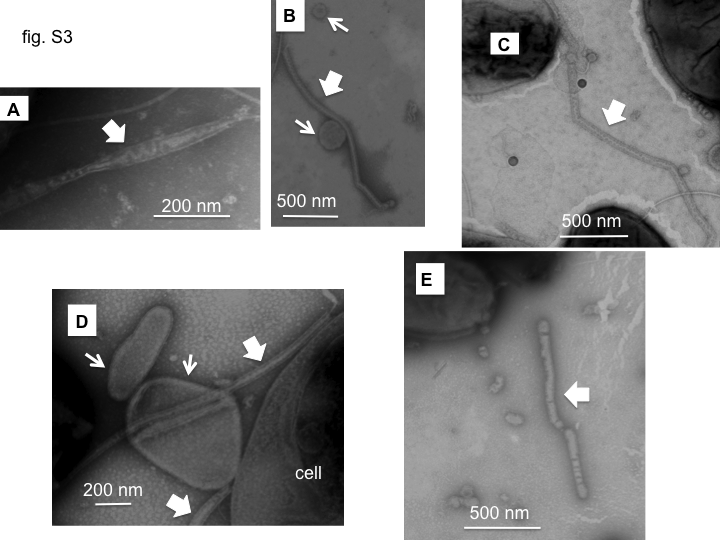

Supplement: Figure S3 — Negatively stained culture fluid samples containing nanopods from other Comamonadaceae bacteria. Nanopods are indicated by thick arrows, thin arrows point to other apparent S-layer-derived structures (indicated by crystalline surface). Samples are from:, A. avenae subsp. citrulli AAC00-1 (Panel A), A. delafieldii (Panel B), D. acidovorans SPH-1 (Panel C), D. acidovorax ATCC 15688 (Panel D) and V. eiseniae EF01-2 (Panel E). (TIF) [file pone.0020725.s003.tif]
